# Early-onset colorectal cancer is associated with metabolic disorders: a systematic review and meta-analysis

**DOI:** 10.1007/s10654-025-01359-x

**Published:** 2026-01-24

**Authors:** Jacques Hilbert, Fernando Topfstedt, Laura Matuschik, Lars Schmitt, Ilaria Panzeri, John Andrew Pospisilik, Gabriel Seifert

**Affiliations:** 1 Department of General and Visceral Surgery, Medical Center - University of Freiburg Medical, Freiburg, Germany; 2 Department of General and Visceral Surgery, Emmendingen Medical Center - University of Freiburg, Emmendingen, Germany; 3 Department of Epigenetics, Van Andel Institute, Grand Rapids, Michigan USA; 4 Max Planck Institute of Immunobiology and Epigenetics, Freiburg, Germany

**Keywords:** Metabolic, Colorectal, Cancer, Early-Onset, Risk factors

## Abstract

Early-onset colorectal cancer (eoCRC), defined by diagnosis before age 50, is increasing worldwide. Metabolic disorders are suspected contributors. We performed a systematic review and meta-analysis to quantify associations between eoCRC and obesity, type 2 diabetes, hyperlipidemia, arterial hypertension and metabolic syndrome. We systematically searched MEDLINE, Cochrane Central Register of systematic reviews, EMBASE, ClinicalTrials.gov, and Web of Science from March 2023 to December 2024. A univariate meta-analysis was performed for outcomes with at least four studies and comparable means of association. 38 studies were included. Obesity at diagnosis was associated significantly with a 1.45-fold increased risk of eoCRC. Elevated BMI during late adolescence, at age 20, and at age 30 were associated with higher eoCRC risk in multiple cohort studies. as independent risk factors. Among male individuals aged 20–49, Type 2 diabetes increased eoCRC risk, with affected individuals exhibiting a 10-year colorectal cancer risk comparable to that of the general population at age 50,but occurring 4–5 years earlier. Additional positive associations were reported for hyperlipidemia (ages 20–39), arterial hypertension in males (ages 20–39), and metabolic syndrome, although findings were heterogeneous. A higher number of metabolic comorbidities was positively correlated with increased eoCRC risk. Early metabolic dysregulation appears to accelerate colorectal carcinogenesis, increasing the impact of metabolic risk factors at younger ages. As metabolic disorders rise among adolescents and young adults, the eoCRC burden likely will grow. Life-course studies integrating metabolic trajectories, molecular biomarkers, epidemiologic data while accounting for screening exposure are needed to clarify causal pathways and guide prevention and screening.

**Supplementary Information:**

The online version contains supplementary material available at 10.1007/s10654-025-01359-x.

## Introduction

Colorectal cancer (CRC) is the third most frequent cancer worldwide with the second highest disease-specific mortality [1]. Recently, early-onset CRC (eoCRC) was distinguished from late-onset colorectal carcinoma (loCRC) based on the age at diagnosis. The cut-off between eoCRC and loCRC is commonly defined as 50 years of age, but other cut-offs between 40 and 55 years have been proposed [2–4]. Despite a decline in loCRC-incidence [5], there has been an increase of eoCRC-incidence in many countries, including the US [5–7], China, Japan [8] and several European countries [9]. Notably, sex bias in colorectal cancer reveals that men exhibit a higher incidence of both loCRC and eoCRC; however, the disparity is more pronounced in loCRC, whereas eoCRC demonstrates a more equal distribution. The mechanisms underpinning the recent surge in eoCRC remain unclear. That said, differences in eoCRC-associated tumor characteristics suggest significant influence of environmental, ethnic and lifestyle factors [10].

Compared to those with loCRC, patients with eoCRC present with more advanced disease [11], at a more distal location, and with worse differentiation state; a higher proportion of eoCRC cases are also linked to hereditary cancer syndromes or prevalence of first-degree relatives with CRC [11, 12]. As of now, there is little evidence on the relative importance of genetics and non-genetic factors in eoCRC compared with loCRC.

With metabolic disorders rising globally among younger populations, understanding their role in eoCRC is imperative. This study provides **a comprehensive meta-analytic quantification of this link**, including associations with obesity, type 2 diabetes (T2D), hyperlipidemia (HLD), arterial hypertension (HTN), and metabolic syndrome (MetS), as well as notable differences across sex and age groups. Taken together, these findings urge the need for tailored screening and intervention strategies [13].

## Materials & methods

We systematically searched for studies in MEDLINE, Cochrane Central Register of Controlled Trials, EMBASE, ClinicalTrials.gov and Web of Science investigating metabolic risk factors and comorbidities associated with incidence of CRC. A particular focus was on obesity, T2D, HLD, HTN and MetS. Search strategies were refined using Ovid MEDLINE. Exact search terms are specified in Supplementary Figure S1. We included studies published in English between January 1st 2010 and December 6th 2024 that included at least 50 eoCRC cases and described any association with metabolic disorders. Initially, all abstracts were screened to avoid specific relevant exclusions and to identify the studies relevant to the above-mentioned research question. Subsequently, full-text screening was conducted by the main searcher (J.H.) to identify papers relevant to the research question. None of the excluded non-English studies met the other inclusion criteria. It should be emphasized that the definition of eoCRC or purely descriptive results were not exclusion criteria for study selection.

Initially, 2015 articles were identified with 253 eligible for full-text review. 38 studies were included in the meta-analysis. Exclusions during full-text review were primarily due to a lack of age stratification or mean age superior to 50 years (*n* = 97), deviation from the topic (*n* = 51), or failure to differentiate between early-onset colorectal neoplasm and carcinoma (*n* = 34) (Supplementary Figure S2: PRISMA flow diagram).

Studies were assessed and subsequently classified by using GRADE (Supplementary Figure S3) [14]. Each step of study exclusion and the assessment of evidence quality was independently reviewed by three co-searchers (F.T., G.S., L.M.). GRADE is a framework for grading the quality of evidence and strength of recommendations applicable to a variety of study designs without adaptation. Five studies had “moderate” evidence, 18 had “low”, and 15 had “very low”. “Moderate” evidence is considered clinically relevant due to strict criteria for observational studies.

Supplementary Figure S4 summarizes the baseline characteristics of 38 studies, totaling 117,361 eoCRC cases. Most were cohort studies (*n* = 23) and conducted in the U.S. (*n* = 20). Elangovan et al. contributed the largest number of cases (*n* = 16,090). Studies adjusted for various covariates including age, race, inflammatory bowel disease (IBD), family history of CRC, smoking, alcohol intake, physical activity, diet, and aspirin use. We extracted information on study type, outcomes measured, study population, methods, statistical analysis and complete results. Unless stated otherwise, we reported multivariable-adjusted effect measures of the individual studies. A comparison of demographics, lifestyle factors, outcome measures and evidence level were performed. Except for five studies, which either only included male or female sex individuals [15–20], all the included studies had a low risk for sex bias. Unless otherwise stated, all results were retrieved from sex-balanced study populations. While some studies specifically examined certain ethnic subgroups or were limited to one ethnic group [19, 21–26], most of the multivariate-adjusted outcomes accounted for both sex and ethnicity.

### Definitions

There is considerable heterogeneity in study designs, populations and granularity of risk factor measures. The following provides a short overview (Supplementary Table S5 for study-specific definitions):

- eoCRC: Diagnosed before age 50.
- Obesity/Overweight: WHO Body mass Index (BMI) cutoffs.
- T2D: ICD codes, HbA1c > 7%, or mixed clinical criteria.
- HLD: ICD codes, lipid profiles; some used sex-specific HDL thresholds.
- HTN: Ranged from BP > 130/85 mmHg to AHA guideline categories.
- MetS: Definitions included NCEP-ATP III, IDF criteria, or medication proxies.

### Statistical analysis

The primary endpoint was to assess associations between eoCRC and metabolic comorbidities. We grouped findings for each metabolic comorbidity by outcome. A forest plot was created for outcomes with at least four comparable studies, converting Odds ratios (OR) to relative risks (RR) where necessary.

In high-incidence populations (> 10%), OR was converted using the formula by Zhang et al. [27]. We performed a meta-analysis for outcomes with four or more studies, pooling estimates of subgroups where applicable. Due to the heterogeneous definitions and measurement methods, we used an inverse variance random-effect model with Knapp-Hartung adjustments. We estimated the heterogeneity variance restricted maximum likelihood (REML) [28] and, although biased in small meta-analyses, I^2^ and t^2^ as heterogeneity statistic [29].

Outcomes with fewer than four studies were described individually, including measures of association and distinct limitations. Statistical analysis and meta-analysis performed using R (Version 4.3.2) and RStudio (Version 2023.09.1) with forest plots generated using the “forestplot” library authored by a co-author F.T.

## Results

### Obesity and overweight

We identified multiple studies analysing obesity-related outcomes: 16 cohort studies, two cross-sectional and six case-control studies.

**Fig. 1:**
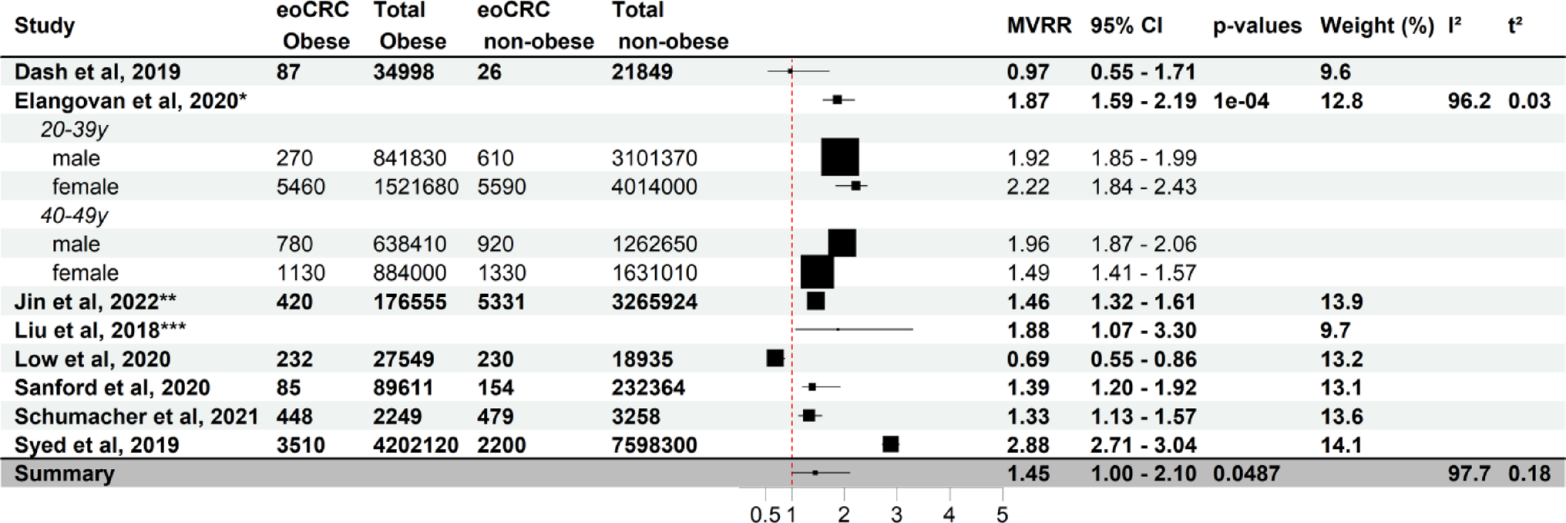
Effect of obesity at diagnosis on eoCRC risk. Forest plot of a meta-analysis of studies analysing the association of obesity at cancer diagnosis and eoCRC (full study names in references). Each study (stratified by age where provided) shows multivariate-adjusted risk ratios (MVRR), number of cases and individuals at risk. The horizontal lines indicate confidence intervals. Boxes indicate estimates of adjusted risk ratios. The size of the boxes inversely correlates with the width of the confidence intervals. The last row shows the pooled adjusted risk ratio. eoCRC Obese: study population with early-onset colorectal cancer and a BMI superior or equal to 30 kg/m^2^. Total Obese: study population with a BMI superior or equal to 30 kg/m^2^. eoCRC non-obese: study population with early-onset colorectal cancer and a BMI inferior to 25 kg/m^2^. Total non-obese: study population with a BMI inferior to 25 kg/m^2^. *Pooled risk ratio estimate from subgroups **: estimation of population numbers by dividing person-years by the median time of follow-up ***Prospective open cohort study only providing person-years as quantifiable size of study population

**Fig. 2:**
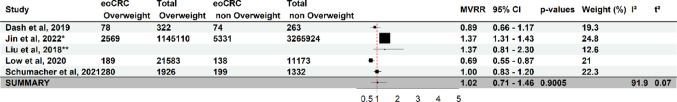
Effect of overweight at diagnosis on eoCRC risk. Forest plot of a meta-analysis of studies analysing the association of overweight at cancer diagnosis and eoCRC (full study names in references). Each study (stratified by age where provided) shows multivariate-adjusted risk ratios (MVRR), number of cases and individuals at risk. The horizontal lines indicate confidence intervals. Boxes indicate estimates of adjusted risk ratios. The size of the boxes inversely correlates with the width of the confidence intervals. The last row shows the pooled adjusted risk ratio. eoCRC Overweight: study population with early-onset colorectal cancer and a BMI between 25 and 29.99 kg/m^2^. Total Overweight: study population with a BMI between 25 and 29.99 kg/m^2^. eoCRC non-overweight: study population with early-onset colorectal cancer and a BMI inferior to 25 kg/m^2^. Total non-overweight: study population with a BMI inferior to 25 kg/m^2^. *Pooled risk ratio estimate from subgroups **: estimation of population numbers by dividing person-years by the median time of follow-up ***Prospective open cohort study only providing person-years as quantifiable size of study population

Most studies investigated the association of obesity at CRC diagnosis with eoCRC. The pooled adjusted risk ratio shows a statistically significant 1.45-fold risk of eoCRC in patients with obesity compared with controls (Summary RR 1.45 [1.00–2.10.00.10]) with extremely high heterogeneity and substantial between-study variance (Fig. 1) [2, 18–21, 23, 26, 30–35]. Being overweight at onset was not statistically associated with risk of eoCRC (Summary RR 1.02 [0.71–1.46]) (Fig. 2) [18–20, 26, 31]. This association shows high heterogeneity with moderate variance. One study found obesity at the time of colonoscopy to be a protective factor (multi-variable adjusted OR (MVOR) 0.69 [0.55–0.86]). However, this study included only US veterans(mostly male) and did not include any data on family history of CRC [20].Compared with loCRC, eoCRC patients were significantly less likely to be overweight (MVOR 0.56 [0.41–0.76]) or obese (MVOR 0.66 [0.48–0.90]) [36].

Four studies investigated obesity before cancer diagnosis with eoCRC. Risk of eoCRC was associated with **A** BMI above the 85th percentile in late adolescence colon cancer for male individuals (Hazard Ratio (HR) 1.53 [1.17–2.00.17.00]); **B** rectal cancer for male individuals (HR 1.09 [0.68–1.73]); **C** BMI ≥ 30 kg/m² at age 20 and significantly at age 30 (MVOR 2.56 [1.18–5.44], 2.06 [1.25–3.40]); **D** each 5-unit increase of the BMI at 20 and significantly at 30 years (MVOR 1.44 [1.18–1.75], 1.36 [1.15–1.61]) with diverging results across studies; and **E** an obese phenotype 10 years before CRC diagnosis (MVOR 1.88 [1.30–2.73]) [4, 16, 17, 37–39].

Two studies examined weight gain since adulthood in female-only cohorts. One found no association, whereas the other reported that weight gain of more than 40 kg (MVRR) 2.15 [1.01–4.55]) and each 5 kg-weight increase since adolescence (MVRR 1.09 [1.02–1.16]) were associated with higher eoCRC risk among women [18, 19].

One study assessed longitudinal obesity and abdominal obesity trajectories and found that maintained overweight over a 2-year period (MVHR 1.09 [1.03–1.16]), and constant abdominal obesity (defined by the Korean Society for the Study of Obesity [40]) (multivariate-adjusted HR (MVHR) 1.18 [1.09–1.29]) were independent risk factors. Neither an increase nor a decrease in BMI or waist circumference during this time frame showed additional influence on eoCRC risk [22].

Only one study reported a lower prevalence of overweight and obesity in eoCRC compared to loCRC (27.8% vs. 37.9% and 30.6% vs. 36.4%), but these differences were not statistically significant [41].

A South Korean study found that higher waist circumference was associated with higher incidence of eoCRC (male < 100 cm/female < 95 cm vs. male < 80 cm/female < 75 cm MVHR 1.28 [1.20–1.37]) [26].

In summary, the literature highlights an association between obesity and eoCRC risk, including progressive associations with early adulthood obesity. Long-term obesity (> 10 years pre-diagnosis) is associated with eoCRC risk, as are substantial weight gain since adolescence, persistent overweight, and abdominal obesity.

### Type 2 diabetes (T2D)

To assess T2D as a risk factor for eoCRC, eighteen studies (ten cohort, seven case-control, one cross-sectional study) were analysed.

Most studies analysing T2D-related outcomes investigated the association between T2D at CRC diagnosis and eoCRC-incidence [2, 3, 20, 21, 23, 25, 26, 30–32, 35, 38, 39, 42, 43].

**Fig. 3:**
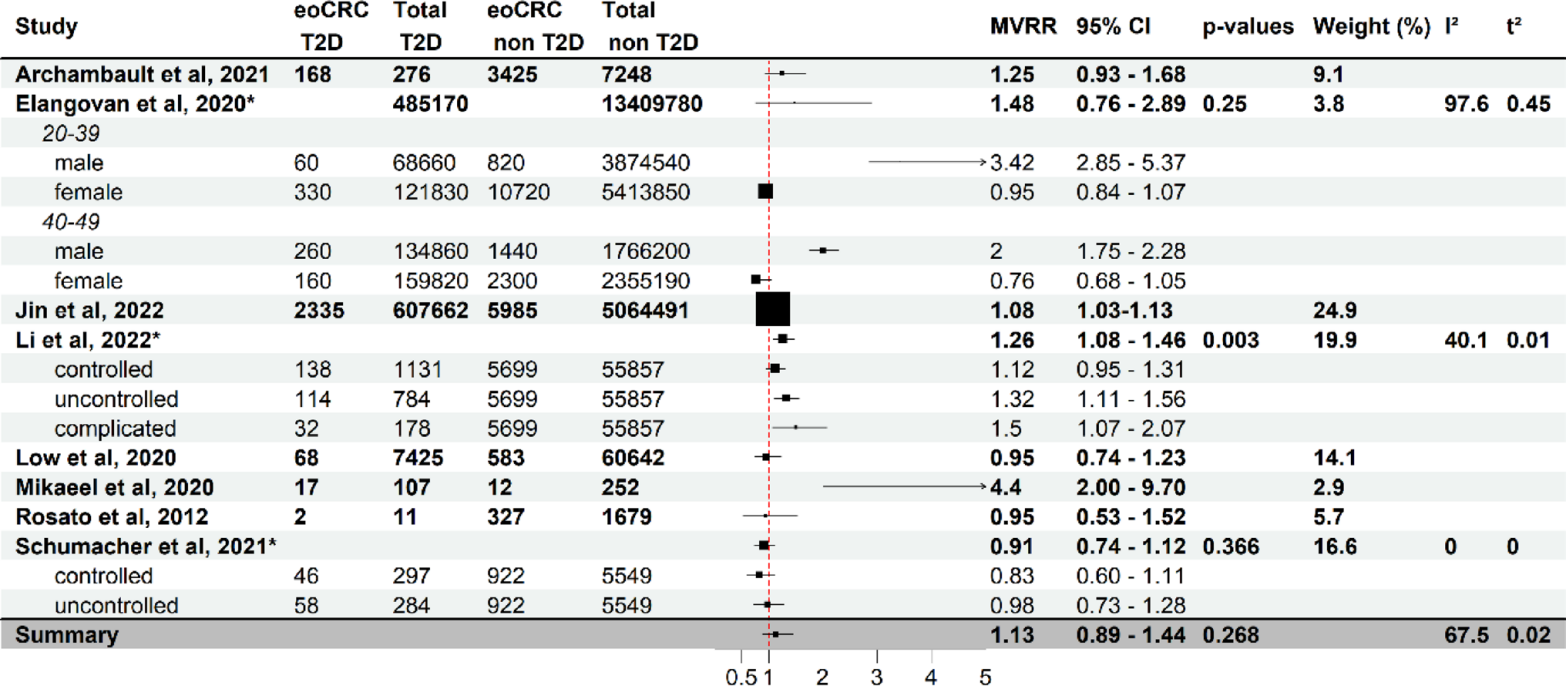
Effect of type 2 diabetes at diagnosis on eoCRC risk. Forest plot of a meta-analysis of studies analysing the association of Type 2 diabetes (T2D) at cancer diagnosis and eoCRC (full study names in references). Each study (stratified by age where provided) shows multivariate-adjusted risk ratios (MVRR), number of cases and individuals at risk. The horizontal lines indicate confidence intervals. Boxes indicate estimates of adjusted risk ratios. The size of the boxes inversely correlates with the width of the confidence intervals. The last row shows the pooled adjusted risk ratio. Only five out of nine outcome-related studies have been used for the forest plot and meta-analysis due to some either using a different measure of association without possibility for conversion or their definition of outcome not being suited for a direct comparison. eoCRC T2D: study population with early-onset colorectal cancer and Type 2 Diabetes at diagnosis. Total T2D: study population with Type 2 Diabetes at diagnosis. eoCRC non T2D: study population with early-onset colorectal cancer without Type 2 Diabetes. Total non T2D: study population without Type 2 Diabetes at diagnosis. *Pooled risk ratio estimate from subgroups

A meta-analysis showed a non-significant trend between T2D at CRC diagnosis and eoCRC risk with a pooled RR of 1.13 [0.89–1.44]) (Fig. 3) with relatively consistent findings across studies (I^2^ = 67.50%, t^2^ = 0.02). Stratification by age and sex, however, revealed T2D as a risk factor in males, doubling risk in 40–49-year-olds (MVRR 2.00 [1.75–2.28]) and showing higher risks in younger patients (20–39-year-olds: MVRR 3.42 [2.85–5.37]). A U.S. case-control study indicated that controlling hyperglycemia and diabetic complications was protective, while uncontrolled or complicated T2D were associated with a higher eoCRC risk (MVRR 1.32 [1.11–1.56] and 1.50 [1.07–2.07]) [4]. However, this finding did not validate in several separate studies [20, 39, 43–45].

A Swedish nationwide cohort study showed a forward shift of 4 to 5 years in the 10-year cumulative risk of CRC for patients with T2D compared to those without. When T2D was combined with a family history of CRC, the risk advancement increased markedly to 12–21 years relative to the general population [46].

In the same cohort, age at T2D diagnosis influenced the risk of developing eoCRC. Individuals diagnosed with T2D at ages 30–39 or 40–49 had significantly higher standardized incidence ratios (SIR) for eoCRC compared to non-diabetic controls (SIR 1.6 [1.1–2.2] and 3.6 [2.8–4.5] respectively). Additionally, T2D diagnosis at any age was associated with an increased risk of eoCRC (SIR 1.9 [1.6–2.3]), while the association for loCRC was less pronounced [47].

Another study comparing two prospective cohorts found that glucose levels above 7 mM were more strongly associated with eoCRC than loCRC for male individuals(defined here as eoCRC < 55 years). They also demonstrated linear dose-response relationship between glucose levels and overall CRC risk [48].

Collectively, current evidence suggests positive association between T2D and eoCRC in several large observational studies, particularly among males aged 20–49, although the overall pooled estimate was not statistically significant. Early-onset T2D elevates eoCRC incidence (SIR up to 3.6), and when combined with a family history of CRC, the cumulative risk is advanced by 12–21 years. Elevated blood glucose levels (> 7 mM) further reinforce this association, showing a strong, dose-dependent association with eoCRC risk in males.

### Hyperlipidemia (HLD)

Eight studies examined the influence of HLD on eoCRC outcome (three cohort studies, four case-control studies, one cross-sectional study), mostly by focusing on the prevalence of HLD at the time of eoCRC diagnosis.

**Fig. 4:**
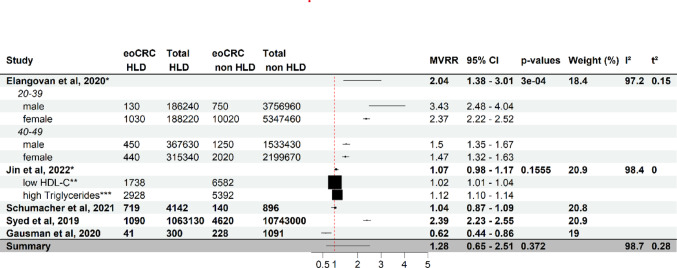
Effect of hyperlipidemia at diagnosis on eoCRC risk. Forest plot of a meta-analysis of studies analysing the association of hyperlipidemia (HLD) at cancer diagnosis and eoCRC (full study names in references). Each study (stratified by age where provided) shows multivariate-adjusted risk ratios (MVRR), number of cases and individuals at risk. The horizontal lines indicate confidence intervals. Boxes indicate estimates of adjusted risk ratios. The size of the boxes inversely correlates with the width of the confidence intervals. The last row shows the pooled adjusted risk ratio. One study is not represented due to it only presenting measures of association for individuals with no hyperlipidemia and eoCRC-incidence [15]. Another is not represented due to it only providing one measure of association for the influence of BMI superior to 30 kg/m^2^ or Type 2 diabetes or hyperlipidemia [32]. eoCRC HLD: study population with early-onset colorectal cancer and hyperlipidemia at diagnosis. Total HLD: study population with hyperlipidemia. eoCRC non HLD: study population with early-onset colorectal cancer and without hyperlipidemia at diagnosis. Total non HLD: study population without hyperlipidemia. *Pooled risk ratio estimate from subgroups. **HDL-c < 50 mg/dL in women, < 40 mg/dL in men. ***serum triglyceride > 150 mg/dL

Multivariate analysis showed conflicting results among studies, with a pooled risk ratio of 1.28 [0.65–2.51], extreme heterogeneity and substantial between-study variance. Larger U.S. and South Korean cohorts have observed a positive independent positive correlation between HLD and risk of eoCRC, especially for 20–39 year olds [26, 30, 34], whereas smaller studies could not confirm these results [15, 31], or argue contrary to the observation [49] (Fig. 4).

### Arterial hypertension (HTN)

Concerning HTN, only its influence on eoCRC incidence has been investigated so far. In total, we identified nine studies that addressed HTN as a potential risk factor for eoCRC [21, 23, 25, 26, 30, 31, 34, 35, 45].

**Fig. 5:**
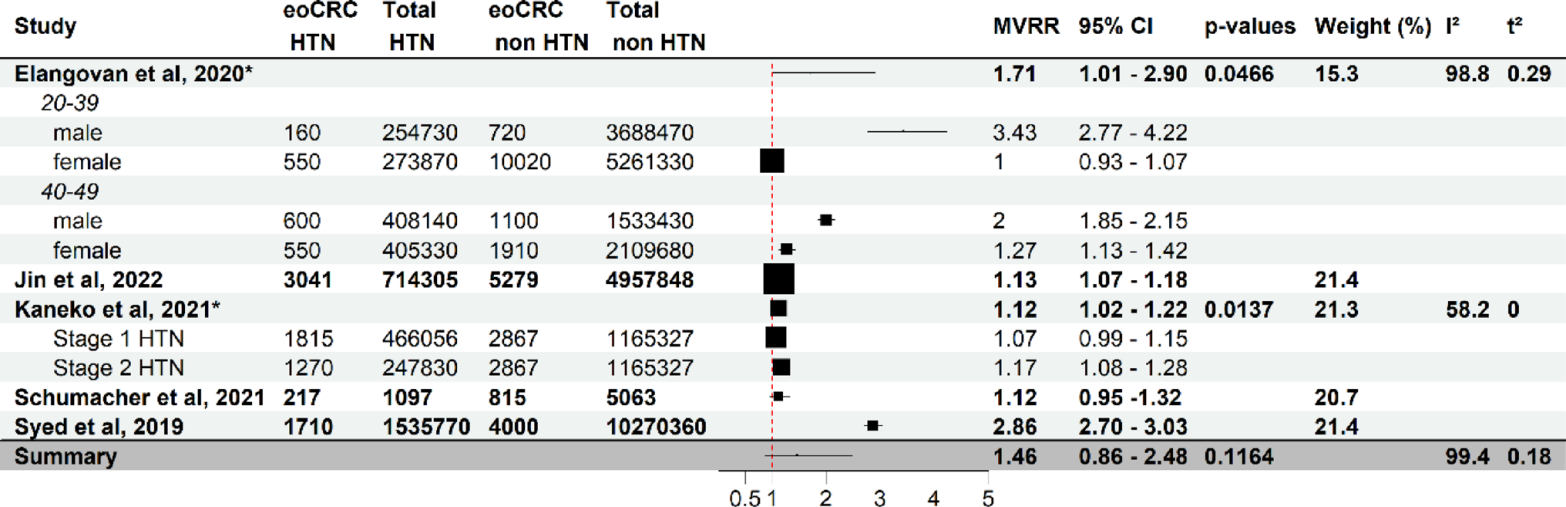
Effect of Arterial hypertension at diagnosis on eoCRC risk. Forest plot of a meta-analysis of studies analysing the association of arterial hypertension (HTN) at cancer diagnosis and eoCRC (full study names in references). Each study (stratified by age where provided) shows multivariate-adjusted risk ratios (MVRR), number of cases and individuals at risk. The horizontal lines indicate confidence intervals. Boxes indicate estimates of adjusted risk ratios. The size of the boxes inversely correlates with the width of the confidence intervals. The last row shows the pooled adjusted risk ratio. One study is not represented due to it using a different measure of association [21]. HTN: arterial hypertension. Stage 1 HTN: systolic pressure 130–139 mmHg or diastolic pressure 80–89 mmHg. Stage 2 HTN: systolic pressure superior to 140 mmHg or diastolic pressure superior to 90 mmHg. eoCRC HTN: study population with early-onset colorectal cancer and arterial hypertension. Total HTN: study population with arterial hypertension, eoCRC non HTN: study population with early-onset colorectal cancer and without arterial hypertension, Total non HTN: study population without arterial hypertension. *Pooled risk ratio estimate from subgroups

The meta-analysis describing hypertension at onset was not statistically significant (summarized risk ratio of 1.46 [0.86–2.48]), had nearly complete heterogeneity and substantial variance., Subgroup analysis demonstrated a strong association between HTN at diagnosis and eoCRC, especially in male patients aged 20–39 and 40–49 years (MVRR 3.43 [2.77–4.22] and 2.00 [1.85–2.15] respectively) [23, 30] (Fig. 5).

A Chinese cohort study found a stronger association in women (MVHR 2.32 [1.01–5.34]) than in men (1.69 [0.60–4.67]), but the authors did not provide a clear definition of arterial hypertension. However, the authors did not provide a clear definition of arterial hypertension [21]. Two U.S. case-control studies failed to replicate this association; in one study, adjustment for family history did not alter the null result [31, 35]. Overall, evidence for an association between hypertension and eoCRC risk is inconsistent and limited.

### Metabolic syndrome (MetS)

Five studies were identified investigating the association between metabolic syndrome or metabolic comorbidities and eoCRC.

Two studies analysed the influence of individual or combined metabolic disorders on eoCRC incidence [24, 50]. Having at least one metabolic comorbidity (overweight/obesity, T2D, HLD and/or HTN) was an independent risk factor for eoCRC incidence (HR 1.82 [1.66–2.00.66.00]). Notably, this risk was most pronounced in individuals with particularly early onset disease: 30–39 year-olds (incidence rate ratio (IRR) 1.83) versus those aged 40–49 (IRR 1.26) and 50–59 (IRR 1.24) [24, 50].

A Chinese study identified metabolic syndrome as an independent risk factor for both eoCRC (MVOR 1.25 [1.098–1.43]) and loCRC (MVOR 1.15 [1.07–1.27]). The risk increased with the number of metabolic comorbidities for eoCRC: one disorder (MVOR 1.09 [1.00–1.17.00.17]), two disorders (MVOR 1.12 [1.01–1.24]), and three disorders (MVOR 1.31 [1.13–1.51]); for loCRC, only three disorders showed an independent association (MVOR 1.22 [1.15–1.29]) [13]. Another study reported progressively larger independent associations per additional metabolic comorbidity for eoCRC (one disorder (MVOR 1.07 [1.01–1.13]), two disorders (MVOR 1.13 [1.06–1.21]), and three disorders (MVOR 1.25 [1.16–1.35], four disorders (MVOR 1.27 [1.15–1.41]), and five disorders (MVOR 1.50 [1.26–1.79]) [26].

In summary therefore, the literature indicates that MetS and its comorbidities arerisk factors for eoCRC, with risk increasing with the number of metabolic disorders and being particularly pronounced in earlier-onset (30–39-year-old)patients or those with multiple comorbidities.

## Survival-related outcomes

In female patients with eoCRC, the presence of multiple metabolic comorbidities was associated with significantly worse long-term survival compared with having a single metabolic comorbidity [44]. Evidence for individual metabolic conditions is heterogeneous: obesity has been reported to markedly worsen cancer-specific survival in one eoCRC cohort (MVHR 11.56 [1.21–110]) [51], but other studies found obesity-associated survival effects only in late-onset cases [52]. HLD appears to confer worse overall survival when present ≥ 5 years before diagnosis [44].

**Fig. 6:**
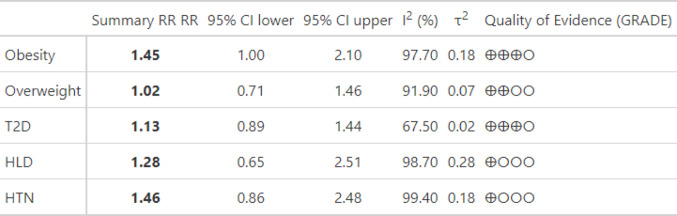
Summary of pooled risk estimates: The table summarizes pooled risk estimates including 95%CI, measures of heterogeneity I^2^ and t^2^ as well as quality of evidence assessment using GRADE. T2D = Type 2 diabetes, HLD = Hyperlipidemia, HTN = Arterial Hypertension, ⊕⊕⊕◯: moderate evidence level, ⊕⊕◯◯: low evidence level, ⊕◯◯◯: very low evidence level

The results of the meta-analyses are summarised in Fig. 6.

## Discussion

Detecting and managing eoCRC remains challenging. Except for individuals with known familial risk or hereditary syndromes, identifying those at elevated risk is difficult. As the prevalence of metabolic comorbidities such as obesity, T2D, MetS increases worldwide—particularly among adolescents and young adults [53, 54]—the incidence of CRC in younger populations is rising in parallel [55–57]. These shifts likely contributed to the rise in eoCRC observed since the 1990 s in the United States and other high-income countries [9, 58, 59]. Global analyses show a steady annual increase in age-standardized eoCRC incidence from 2000 to 2021 [56], and counterfactual models suggest that excess body weight could explain a substantial portion of this increase [23].

Epidemiologic studies reveal an important age-related pattern: eoCRC patients generally show lower absolute prevalence of obesity, T2D, HTN, and HLD at diagnosis compared with loCRC. Himbert et al. reported lower odds of being overweight or obese in eoCRC versus loCRC (OR ≈ 0.56 and 0.66) [36], and Elangovan et al. found fewer metabolic comorbidities in those aged 20–49 years versus 50–74 years [30]. Yet, several studies point to early-life exposures as particularly relevant. Elangovan further noted that obesity was twice as prevalent among eoCRC patients compared with the general population, with the strongest association in women aged 20–39 years. Levi et al., Liu et al., and Li et al. linked late-adolescent obesity to higher eoCRC risk but not loCRC [4, 16–18]; Sanford et al. (predominantly white nurse cohort) observed obesity associated only with eoCRC [33]; and Chen et al. (nested case–control) found that having one or two metabolic comorbidities predicted eoCRC but not loCRC [23].

Associations with T2D are more mixed, though Ali Khan et al. suggested that diabetes may shift the age at which individuals reach population-level CRC risk by several years [47]. Because metabolic conditions accumulate with age and older adults undergo more screening, crude prevalence comparisons between eoCRC and loCRC can be misleading. Disentangling etiologic differences requires life-course exposure data, age-as-timescale modeling, lagged sensitivity analyses to reduce reverse causation, and adjustment for screening behavior. Even with these caveats, converging evidence indicates that metabolic comorbidities may confer a relatively greater proportional risk for eoCRC than for loCRC, suggesting that early-life metabolic dysregulation can accelerate carcinogenesis rather than act solely as a cumulative lifetime hazard [4, 16, 18, 23, 30, 33, 60].

The observed associations are biologically plausible. Metabolic dysregulation involves insulin resistance, chronic inflammation, and altered gut–liver signaling. Elevated insulin and IGF-1 promote epithelial proliferation and inhibit apoptosis, while proinflammatory cytokines (IL-6, TNF-α, CRP) induce oxidative stress and DNA damage [55, 61]. Obesity-related changes in microbiota, bile acid metabolism, and intestinal permeability may further foster a pro-tumorigenic milieu, affecting tumorigenesis and hepatic metastasis [62]. These effects could be amplified when exposures occur earlier in life, during periods of active growth and hormonal change, thus shortening latency to tumor initiation. Sex-specific differences have been reported: among adults aged 20–39 years, obesity appears more strongly associated with eoCRC in women [30], whereas other metabolic comorbidities show stronger associations in men [30, 48]; these differences diminish by age 40–49 years. Across age groups, higher body mass index and greater T2D severity correlate with progressively higher eoCRC incidence, supporting a dose–response relationship [4, 31].

This meta-analysis reinforces evidence linking metabolic comorbidities to CRC risk, but causality cannot be inferred. Residual confounding from lifestyle factors (diet, alcohol, physical activity, family history) likely contributes to heterogeneity across studies. Differential healthcare contact among patients with chronic conditions may lead to detection bias, and BMI measured near diagnosis can be affected by preclinical disease-related weight loss. Prospective studies with standardized exposure assessment, lagged analyses, and explicit adjustment for healthcare utilization are needed.

Limitations include heterogeneity in design, populations, and definitions of both age cut-offs and metabolic exposures. Ethnicity-specific thresholds limit generalizability, and dichotomous coding of comorbidities yields crude effect estimates. Publication bias was judged low, though funnel asymmetry tests were unreliable due to small study numbers (Supplementary Figure S6). Some studies were of modest quality, introducing potential residual confounding. Evidence quality was evaluated using GRADE, risk of bias using ROBIS [63], indicating overall low risk of bias (Supplementary Figure S7). Because studies exclusively addressing eoCRC were not excluded, distinguishing general CRC risk factors from those specific to eoCRC remains difficult.

## Conclusion

In summary, early metabolic dysregulation may act as an accelerator of colorectal carcinogenesis, increasing the relative impact of metabolic risk factors at younger ages. As metabolic disorders become more prevalent among adolescents and young adults, the eoCRC burden is likely to rise further. Integrative, life-course–oriented studies combining early metabolic trajectories, molecular biomarkers, and epidemiologic data—while accounting for screening exposure—are needed to clarify causal pathways and inform targeted prevention and screening strategies.

## Supplementary Information

Below is the link to the electronic supplementary material.

Supplementary Material 1

## Data Availability

Data, analytic methods and study materials will be made available to other researchers on request to the first author.
