# Supplementary material for: Early-onset colorectal cancer is associated with metabolic disorders: a systematic review and meta-analysis
Source: Eur J Epidemiol. 2026 Jan 24;41(3):297–307. doi: 10.1007/s10654-025-01359-x (PMC13222287; doi:10.1007/s10654-025-01359-x)
Supplement: Supplementary file 1 — Supplementary Material 1 [file 10654_2025_1359_MOESM1_ESM.docx]

Supplementary Appendix:

Contents

[Supplementary Figure S1: Research Strategy 2](#_Toc213006935)

[MEDLINE 2](#_Toc213006937)

[WebofScience: 3](#_Toc213006938)

[Cochrane Library: 3](#_Toc213006939)

[Clinicaltrials.gov: 3](#_Toc213006940)

[EMBASE 4](#_Toc213006941)

[Supplementary Figure S2: PRISMA flow diagram 5](#_Toc213006942)

[Supplementary Figure S3: GRADE 6](#_Toc213006943)

[Supplementary Figure S4: Included Trials 7](#_Toc213006944)

[Supplementary Table S5: Exposure definitions 9](#_Toc213006945)

[Supplementary Figure S6: Assessment of publication bias 18](#_Toc213006946)

[Supplementary Figure S7: Risk of Bias assessment using ROBIS 23](#_Toc213006947)

# Supplementary Figure S1: Research Strategy

All search results are limited to a time period between January 1^st^ 2010 and December 6^th^ 2024.

MEDLINE:

| 1 | Insulin/ or Blood Glucose/ or Metabolic Syndrome/ or Diabetes Mellitus/ or Body Weight/ or Hypertension or Dyslipidemia/ | 904137 |
| --- | --- | --- |
| 2 | Colorectal Neoplasms/co, di, ep, et, ge, hi, me, mo, sc, su, th [Complications, Diagnosis, Epidemiology, Etiology, Genetics, History, Metabolism, Mortality, Secondary, Surgery, Therapy] | 87617 |
| 3 | "50".mp. | 1653952 |
| 4 | "Age of Onset"/ or early-onset.mp. or Middle Aged/ | 4971473 |
| 5 | young.mp. | 1595268 |
| 6 | 3 or 4 or 5 | 7088843 |
| 7 | 1 and 2 and 6 |  |
| 8 | age.mp. | 10441985 |
| 9 | 6 or 8 | 12421337 |
| 10 | 1 and 2 and 9 | 558 |
| 11 | limit 10 to yr="2010 -Current" | 434 |

| 1 | Obesity/ or Obesity, Abdominal/ or Obesity, Morbid/ or Overweight/ | 259635 |
| --- | --- | --- |
| 2 | Colorectal Neoplasms/co, di, ep, et, ge, hi, me, mo, sc, su, th | 87406 |
| 3 | "50".mp. | 1423425 |
| 4 | "Age of Onset"/ or early-onset.mp. or Middle Aged/ | 4954402 |
| 5 | young.mp. | 1497689 |
| 6 | age.mp. | 10007022 |
| 7 | 3 or 4 or 5 or 6 | 11731558 |
| 8 | 1 and 2 and 7 | 501 |
| 9 | limit 8 to yr="2010 -Current" | 422 |

## WebofScience:

| 1 | AB = (Colorectal Neoplasm OR Colorectal Neoplasm OR Neoplasm, Colorectal OR Neoplasms, Colorectal OR Colorectal Tumors OR Colorectal Tumor OR Tumor, Colorectal OR Tumors, Colorectal OR Colorectal Cancer OR Cancer, Colorectal OR Cancers, Colorectal OR Colorectal Cancers OR Colorectal Carcinoma OR Carcinoma, Colorectal OR Carcinomas, Colorectal OR Colorectal Carcinomas) | 155446 |
| --- | --- | --- |
| 2 | AB = (Insulin OR Blood Glucose OR Metabolic OR Diabetes Mellitus OR Hypertension) | 1456232 |
| 3 | AB = (50 OR early-onset OR young OR early onset) | 3763199 |
| 4 | 1 and 2 and 3 | 678 |
| 5 | limit 8 to yr="2010 -Current" | 573 |

| 1 | AB = (Colorectal Neoplasm OR Colorectal Neoplasm OR Neoplasm, Colorectal OR Neoplasms, Colorectal OR Colorectal Tumors OR Colorectal Tumor OR Tumor, Colorectal OR Tumors, Colorectal OR Colorectal Cancer OR Cancer, Colorectal OR Cancers, Colorectal OR Colorectal Cancers OR Colorectal Carcinoma OR Carcinoma, Colorectal OR Carcinomas, Colorectal OR Colorectal Carcinomas) | 155446 |
| --- | --- | --- |
| 2 | AB = (Obesity) | 289753 |
| 3 | AB = (50 OR early-onset OR young OR early onset) | 3763199 |
| 4 | 1 and 2 and 3 | 393 |
| 5 | limit 8 to yr="2010 -Current" | 347 |

## Cochrane Library:

| 1 | Colorectal AND Colon AND Rectum:ti,ab,kw | 27 |
| --- | --- | --- |
| 2 | Cancer OR Neoplasm OR Tumor:ti,ab,kw | 1293 |
| 3 | Obesity OR Insulin OR Blood Glucose OR Metabolic OR Diabetes Mellitus OR Hypertension:ti,ab,kw | 1034 |
| 4 | 1 and 2 and 3 | 99 |
| 5 | limit 8 to yr="2010 -Current" | 77 |

## Clinicaltrials.gov:

*Colorectal Neoplasms AND (Metabolic OR Diabetes OR Hypertension OR Obesity OR Insulin OR Blood Glucose OR Hyperlipidemia)*

- **246 Records (Only completed studies) in Clinicaltrials.gov**

EMBASE:

Research strategy did not include any additional limiting for “early” or “young”

*('colorectal tumor' OR 'early onset colorectal cancer'):ti,ab,kw AND ((Obesity OR 'diabetes mellitus' OR 'non insulin dependent diabetes mellitus' OR 'metabolic syndrome X' OR hypertension OR dyslipidemia OR hyperlipidemia OR 'glucose blood level' OR insulin):ab,ti)*

#
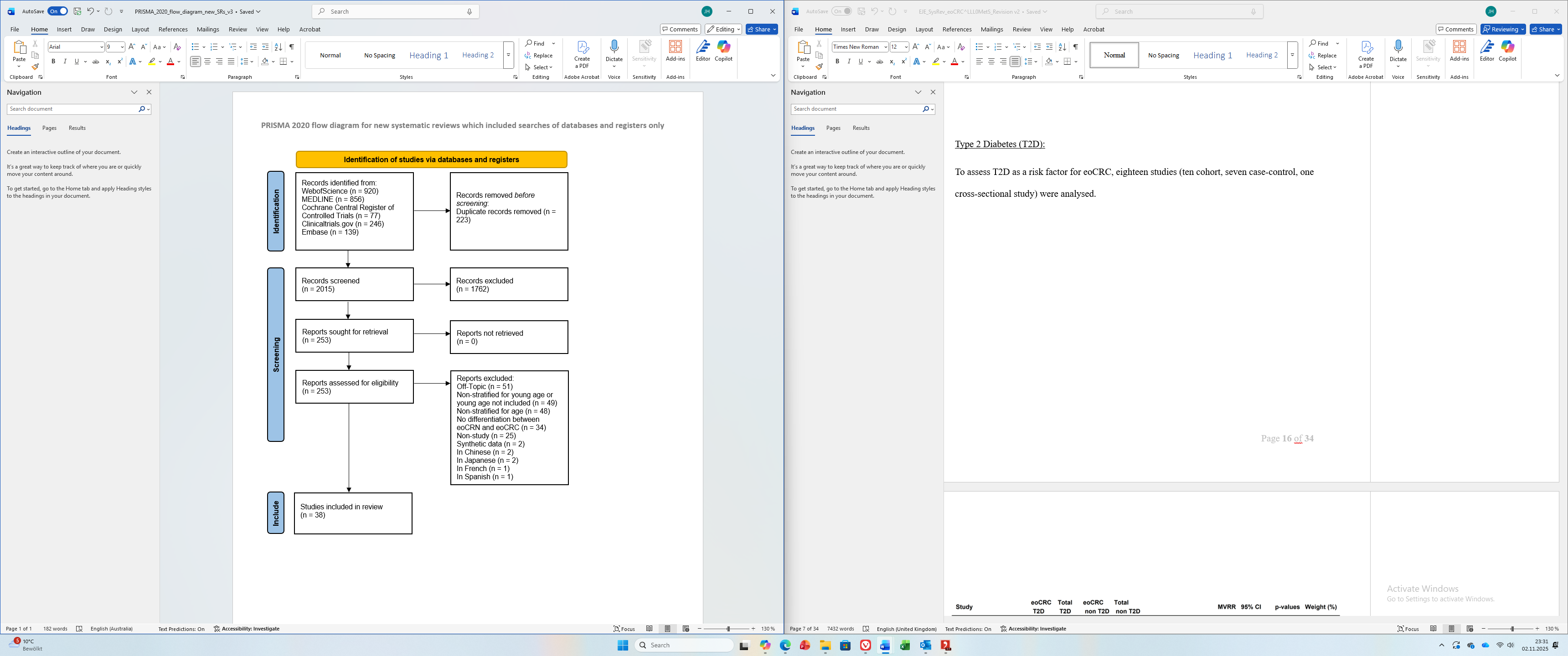
Supplementary Figure S2: PRISMA flow diagram

**Figure S2** **PRISMA flow diagram [1]**

*eoCRN: early-onset colorectal neoplasm (includes carcinoma and adenoma)*

*eoCRN and eoCRC: includes only eoCRN without stratifying for eoCRC*

# Supplementary Figure S3: GRADE


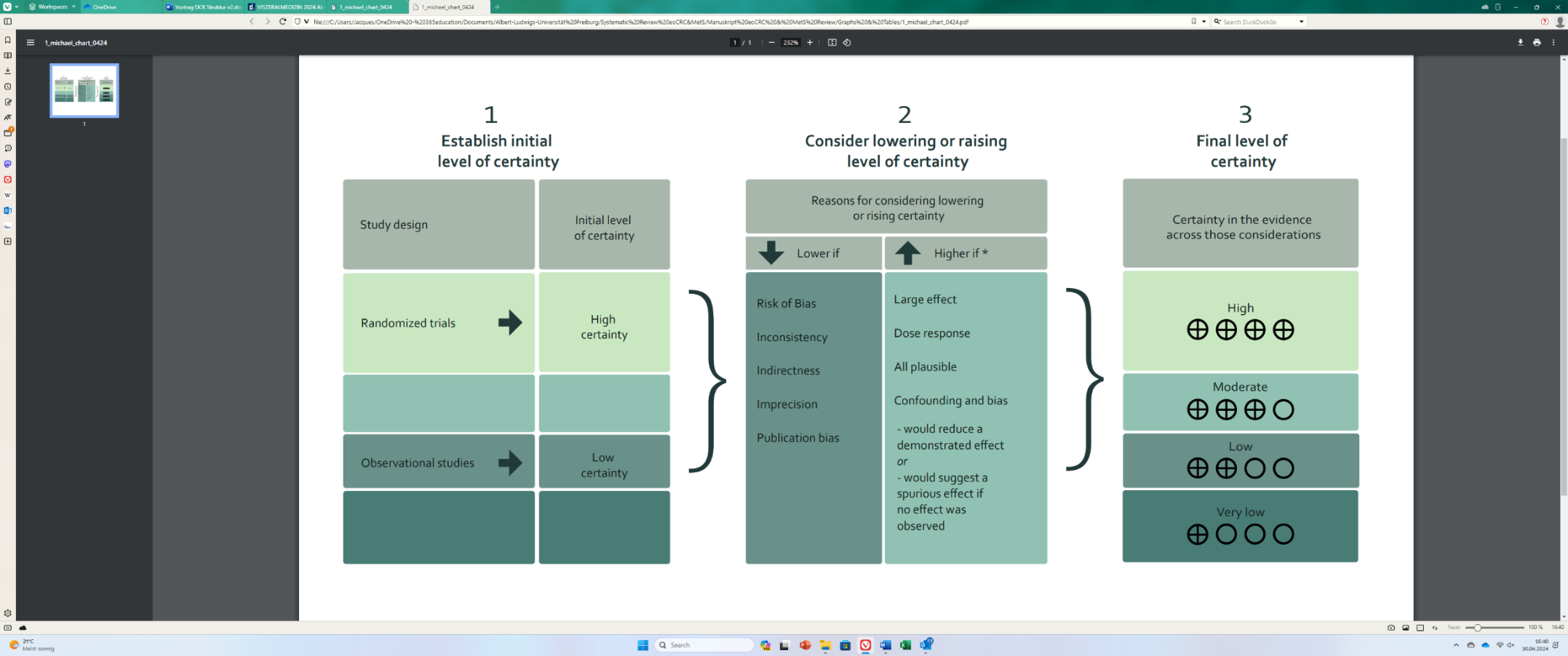


**FIGURE S3** ***Certainty of Evidence Assessment using GRADE****: Evaluating the level of certainty in scientific studies. This process is divided into three main steps:* ***Establish Initial Level of Certainty:****Studies can be characterised into two types: randomized trials and observational studies. The initial certainty for the former is “high”, while for the latter it is “low”.* ***Consider Lowering or Raising Level of Certainty:****Factors that could potentially lower or raise the level of certainty. Lowering factors include risk of bias, inconsistency, indirectness, imprecision, and publication bias. Raising factors include large effect, dose response, and all plausible confounding and bias would reduce a demonstrated effect or suggest a spurious effect if no effect was observed. * Level of Certainty can only be raised if there is no lowering factor.* ***Final Level of Certainty Rating:****Ratings are categorized as “high”, “moderate”, “low”, or “very low”[2]*

# Supplementary Figure S4: Included Trials


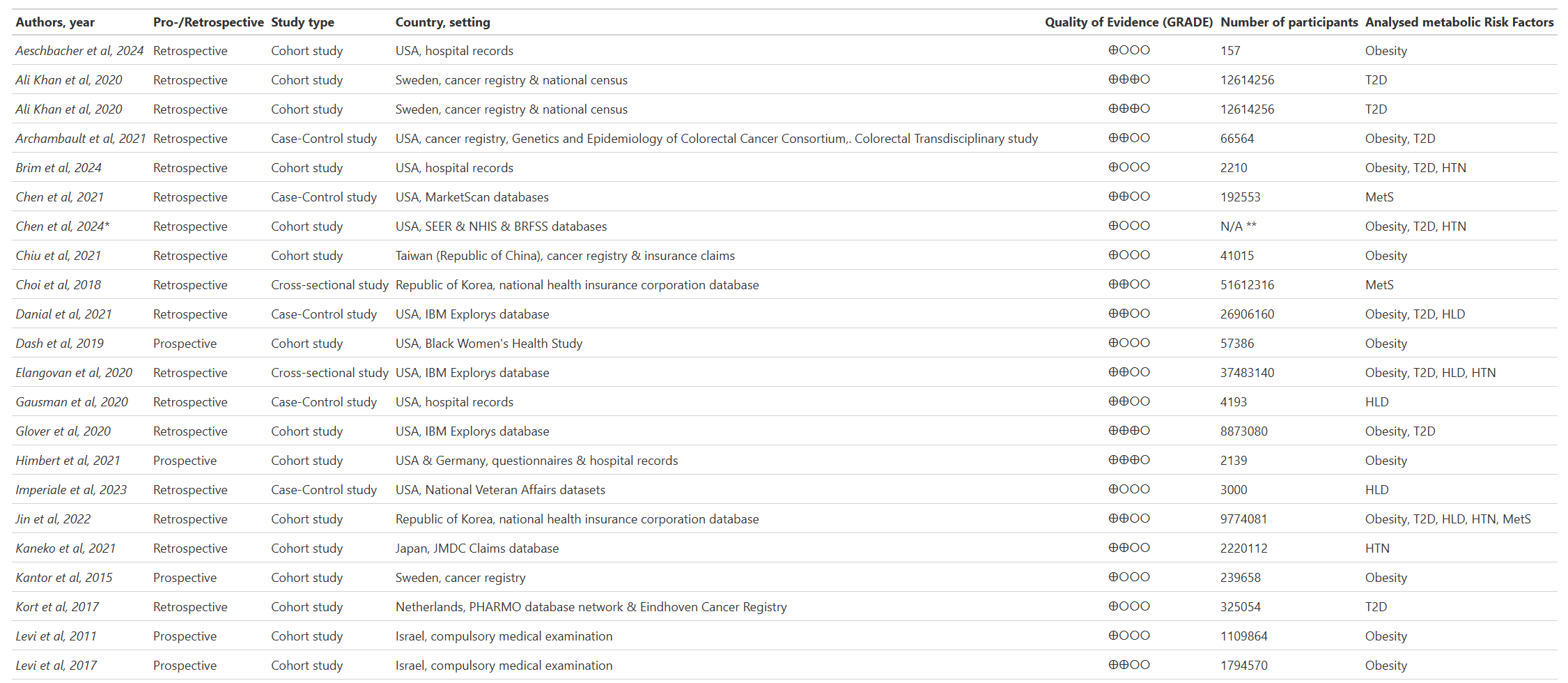


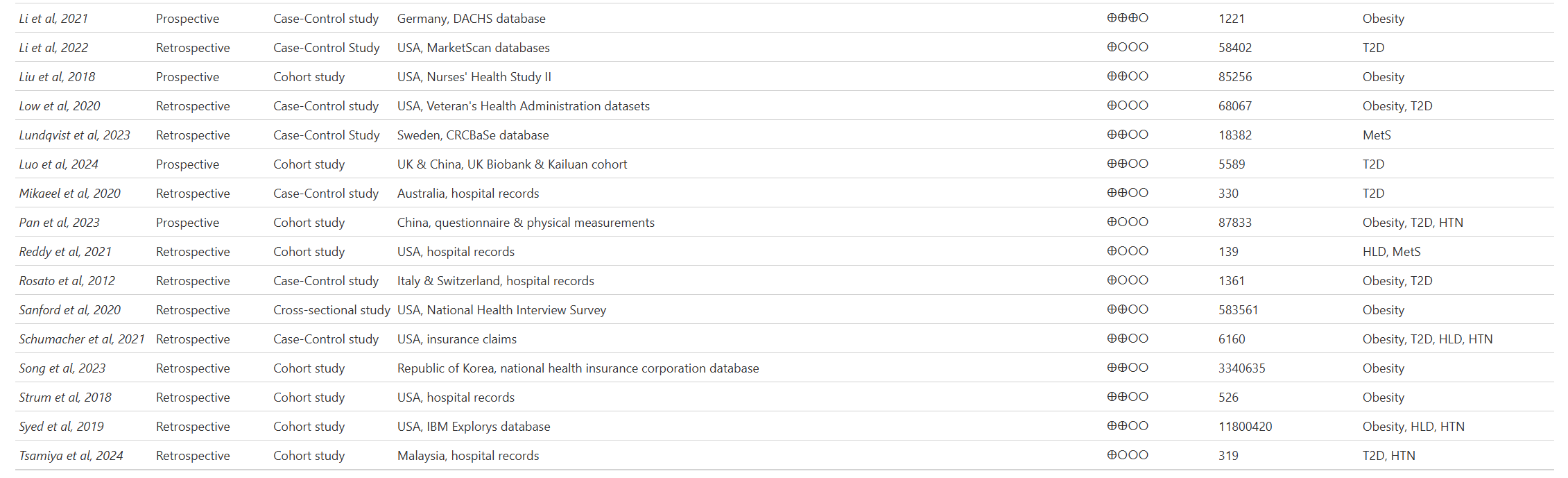


**TABLE S4 *Characteristics of included Trials****: The table summarizes authors, study type, publication year, country of study population, number of study participants, quality of evidence for the respective outcome and analysed risk factors.*

*T2D = Type 2 diabetes, HLD = Hyperlipidemia, HTN = Arterial Hypertension, MetS = Metabolic Syndrome,* ⴲⴲⴲⵔ*: moderate evidence level,* ⴲⴲⵔⵔ*: low evidence level,* ⴲⵔⵔⵔ*: very low evidence level*

**Combination of many different study populations into one study*

*** used patient-years*

# Supplementary Table S5: Exposure definitions

| **Authors, year** | **eoCRC** | **Overweight** | **Obesity** | **T2D** | **HLD** | **HTN** | **MetS** |
| --- | --- | --- | --- | --- | --- | --- | --- |
| *Aeschbacher et al, 2024* | <50y | BMI 25-29.99 kg/m² | BMI ≥30 kg/m² | N/A | ICD codes | ICD codes | N/A |
| *Ali Khan et al, 2020* | <50y | N/A | N/A | ICD codes | N/A | N/A | N/A |
| *Ali Khan et al, 2020* | <50y | N/A | N/A | ICD codes | N/A | N/A | N/A |
| *Archambault et al, 2021* | <50y | N/A | BMI as categorical variable per 5kg/m² | ICD codes | N/A | N/A | N/A |
| *Brim et al, 2024* | <45y | BMI ≥25 kg/m²* | N/A | ICD codes | N/A | ICD codes | N/A |
| *Chen et al, 2021* | <50y | N/A | N/A | N/A | N/A | N/A | NCEP-ATP III: at least 3 of the following: - BMI ≥ 27 kg/m² - Triglyceride ≥ 150 mg/dL or antilipidemic therapy for at least 90 days - HDL <40mg/dL ♂ or <50mg/dL ♀ or drug treatment for low HDL - Blood pressure ≥130/85 mmHg or antihypertensive therapy (>90d) - Fasting serum glucose ≥110 mg/dL or antidiabetic therapy (>90d) |
| *Chen et al, 2024* | <50y | BMI 25-29.99 kg/m² | BMI ≥30 kg/m² | National survey | N/A | National survey | N/A |
| *Chiu et al, 2021* | <50y | BMI 25-29.99 kg/m² | Class I: BMI 30-34.99 kg/m² Class II: ≥35 kg/m² | ICD codes | N/A | N/A | N/A |
| *Choi et al, 2018* | N/A | N/A | N/A | ≥1 antidiabetic medication reimbursement | ≥1 antihyperlipidemic medication reimbursement | ≥1 antihypertensive medication reimbursement | All 3 of the following: - ≥1 antidiabetic medication reimbursement - ≥1 antihyperlipidemic medication reimbursement - ≥1 antihypertensive medication reimbursement |
| *Danial et al, 2021* | <50y | N/A | BMI ≥30 kg/m² | ICD codes | ICD codes | N/A | N/A |
| *Dash et al, 2019* | N/A | BMI 25-29.99 kg/m² | BMI ≥30 kg/m² | N/A | N/A | N/A | N/A |
| *Elangovan et al, 2020* | <50y | N/A | BMI ≥30 kg/m² | ICD codes | ICD codes | ICD codes | N/A |
| *Gausman et al, 2020* | <50y | BMI as a continuous variable | N/A | ICD codes | ICD codes | ICD codes | N/A |
| *Glover et al, 2020* | 20-39y | N/A | BMI ≥30 kg/m² | ICD codes | ICD codes | ICD codes | N/A |
| *Himbert et al, 2021* | <50y | BMI 25-29.99 kg/m² | BMI ≥30 kg/m² | N/A | N/A | N/A | N/A |
| *Imperiale et al, 2023* | <50y | BMI as a continuous variable | N/A | N/A | ICD codes | N/A | N/A |
| *Jin et al, 2022* | <50y | N/A | Obese: 25-29.9 kg/m² Severely obese: ≥30 kg/m²** Abdominal obesity: Waist circumference ≥90cm ♂, ≥95cm ♀ | fasting plasma glucose >100 mg/dL or antidiabetic therapy | HDL-C: serum HDL-C <40mg/dL ♂ or <50mg/dL ♀  Triglycerides: serum triglyceride >150mg/dL | >130/85 mmHg or antihypertensive therapy | At least 3 of the following: - Waist circumference ≥90cm ♂, ≥95cm ♀ - fasting plasma glucose >100 mg/dL or antidiabetic therapy - blood pressure >130/85 mmHg or antihypertensive therapy - serum HDL-C <40mg/dL ♂ or <50mg/dL ♀  - serum triglyceride >150 mg/dL |
| *Kaneko et al, 2021* | N/A | N/A | BMI as a continuous variable | ICD codes | ICD codes | Stage 1: 130-139 mmHg systolic pressure or 80-89 mmHg diastolic pressure Stage 2: ≥140 mmHg systolic pressure or ≥90 mmHg diastolic pressure | N/A |
| *Kantor et al, 2015* | N/A | lower overweight: BMI 25-27.5 kg/m² upper overweight: BMI 27.5-29.9 kg/m² | BMI ≥30 kg/m² | N/A | N/A | N/A | N/A |
| *Kort et al, 2017* | <55y | N/A | N/A | use of antidiabetic medication | N/A | N/A | N/A |
| *Levi et al, 2011* | N/A | - adolescence:  BMI ≥85th percentile - adult: BMI ≥ 25 kg/m²* | N/A | N/A | N/A | N/A | N/A |
| *Levi et al, 2017* | N/A | - adolescence:  BMI 85th -95th percentile - adult: BMI ≥ 25-29.9 kg/m² | - adolescence:  BMI ≥95th percentile - adult: BMI ≥30 kg/m² | N/A | N/A | N/A | N/A |
| *Li et al, 2021* | <50y | BMI 25-29.99 kg/m² | BMI ≥30 kg/m² | N/A | N/A | N/A | N/A |
| *Li et al, 2022* | <50y | N/A | ICD codes | ICD codes (controlled, uncontrolled, complicated) | N/A | N/A | N/A |
| *Liu et al, 2018* | <50y | BMI 25-29.99 kg/m² | BMI ≥30 kg/m² | N/A | N/A | N/A | N/A |
| *Low et al, 2020* | <50y | BMI 25-29.99 kg/m² | BMI ≥30 kg/m² | ICD codes | N/A | N/A | N/A |
| *Lundqvist et al, 2023* | <50y | ICD codes | ICD codes | ICD codes | ICD codes | ICD codes | N/A |
| *Luo et al, 2024* | <55y | N/A | N/A | Blood glucose >7 mmol/L | N/A | N/A | N/A |
| *Mikaeel et al, 2020* | <55y | N/A | N/A | admission interview, ICD codes, medication history | N/A | N/A | N/A |
| *Pan et al, 2023* | <50y | BMI 24-27.99 kg/m²*** | BMI ≥28 kg/m²*** | admission interview (no clear criteria provided) | N/A | admission interview (no clear criteria provided) | N/A |
| *Reddy et al, 2021* | <50y | BMI 25-29.99 kg/m² | Obese: BMI 30-39.99 kg/m² Morbidly obese: ≥BMI 40 kg/m² | HbA1c > 6.5% | low-density lipoprotein (LDL) > 160 mg/dL | at least two ambulatory BP readings > 129/89 mmHg | N/A |
| *Rosato et al, 2012* | <45y | BMI ≥25 kg/m²* | N/A | admission interview | N/A | N/A | N/A |
| *Sanford et al, 2020* | <50y | N/A | BMI ≥30 kg/m² | N/A | N/A | N/A | N/A |
| *Schumacher et al, 2021* | <50y | BMI 25-29.99 kg/m² | BMI ≥30 kg/m² | ICD codes, medication use, laboratory test results controlled: <7% uncontrolled: ≥7% | abnormal test results for LDL, HDL, total cholesterol or triglycerides | ICD codes and antihypertensive medication | N/A |
| *Song et al, 2023* | <50y | N/A | BMI ≥25 kg/m²** Abdominal obesity: Waist circumference >90cm ♂ and ≥ 85cm ♀ | fasting glucose ≥126mg/dL or history of antidiabetic medication | total cholesterol level ≥240mg/dL after a 12-h fast or history of receiving dyslipidaemia medication | >140/90 mmHg or history of receiving antihypertensive medications | 3 or more of the following 5 criteria were satisfied:  - Waist Circumference ≥90cm ♂, ≥85cm ♀  - blood pressure ≥ 130/85mmHg or history of antihypertensive medication  - fasting plasma glucose ≥100mg/dL or history of antidiabetic medication  - serum triglyceride level ≥ 150mg/ dL or drug treatment for elevated triglycerides  - HDL-C <40 mg/dL ♂, <50mg/dL ♀ |
| *Strum et al, 2018* | <50y | BMI 25-29.99 kg/m² | BMI ≥30 kg/m² | N/A | N/A | N/A | N/A |
| *Syed et al, 2019* | <50y | N/A | BMI ≥30 kg/m² | N/A | ICD codes | ICD codes | N/A |
| *Tsamiya et al, 2024* | <50y | N/A | N/A | N/A | ICD codes | ICD codes | N/A |

**TABLE S5 *Exposure definitions****: The table summarizes exposure definitions for eoCRC, Overweight, Obesity, T2D, HLD, HTN and MetS*

**No differentiation in between overweight and obesity*

*** WHO recommendations for Asia-Pacific region [3]*

****Chinese standard for obesity according to Chinese Center for Disease Control and Prevention*

# Supplementary Figure S6: Assessment of publication bias

| Obesity | 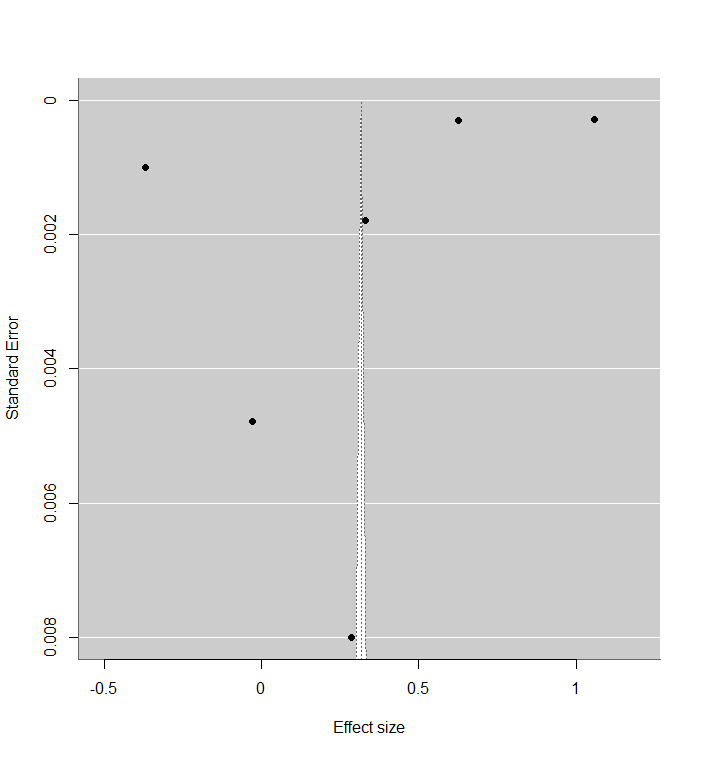*(only including studies assessing number of patients, excluding studies assessing patient years)*  Trim-and-fill estimated no missing studies and produced an adjusted pooled OR identical to summary RR (with the omission of Liu et al, which reported patient-years), and Egger’s test for funnel-plot asymmetry was non-significant (z = -0.62, p = 0.54). |
| --- | --- |
| Overweight | Trim-and-fill estimated no missing studies and produced an adjusted pooled OR identical to summary RR (with the omission of Liu et al). Egger’s test was non-significant (z = −0.29, p = 0.77). 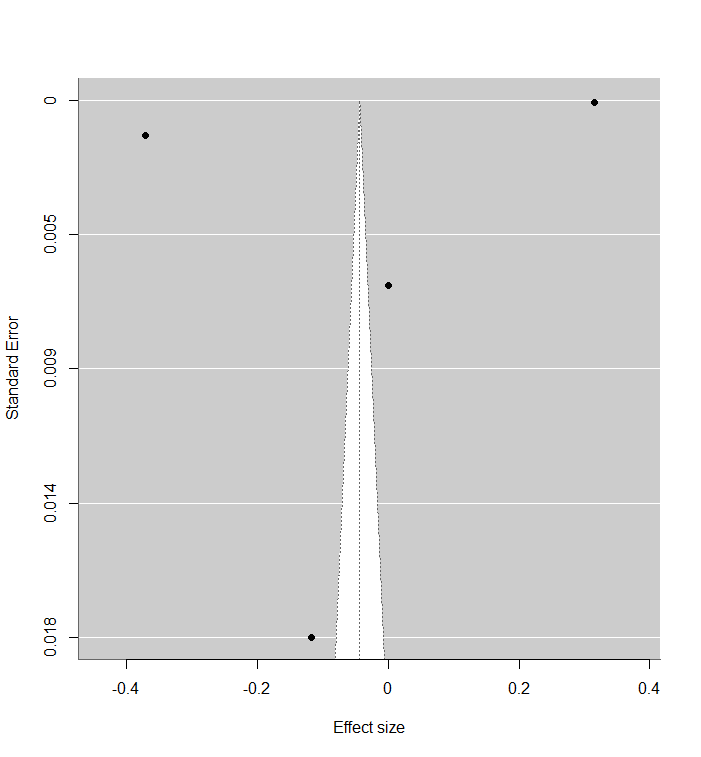 |
| T2D | *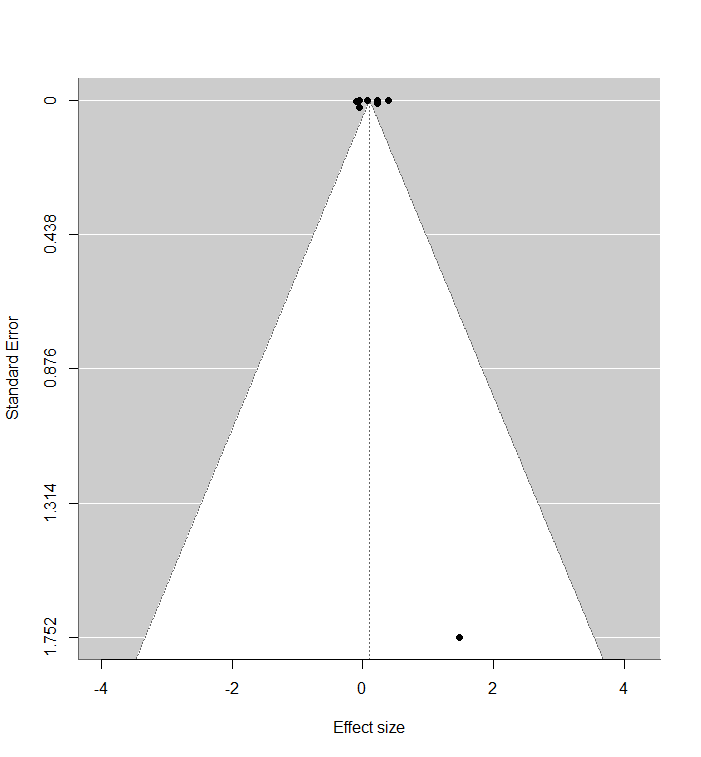(only including studies assessing number of patients, excluding studies assessing patient years)*  Trim-and-fill estimated one potentially missing study on the left, resulting in an adjusted pooled RR of 1.11 (95% CI 0.97–1.27). Egger’s test for funnel-plot asymmetry was non-significant (z = 0.70, p = 0.49).  *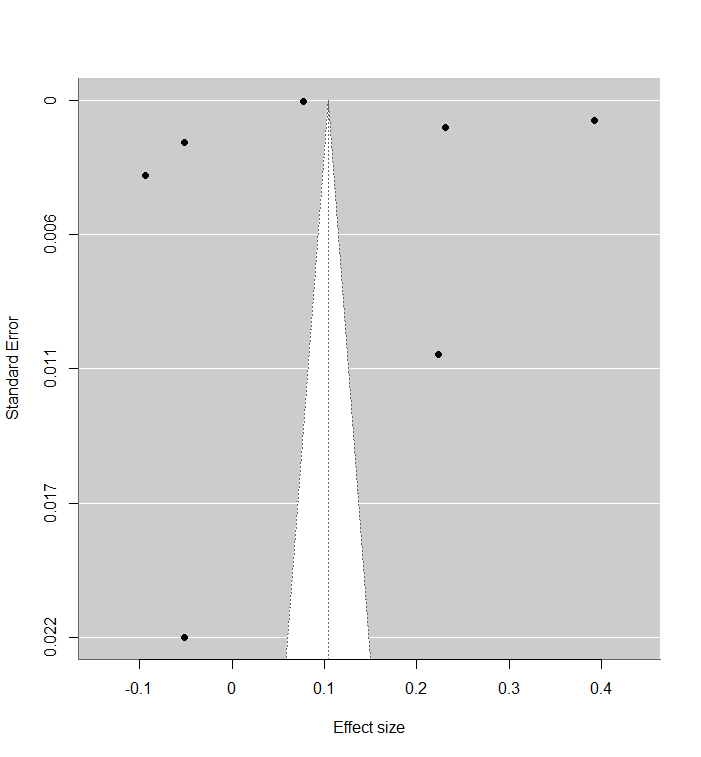*  Trim-and-fill estimated no missing studies and produced an identical pooled RR (after having omitted Liu et al and Mikaeel et al); Egger’s test was non-significant (z = −0.70, p = 0.48). |
| HLD | *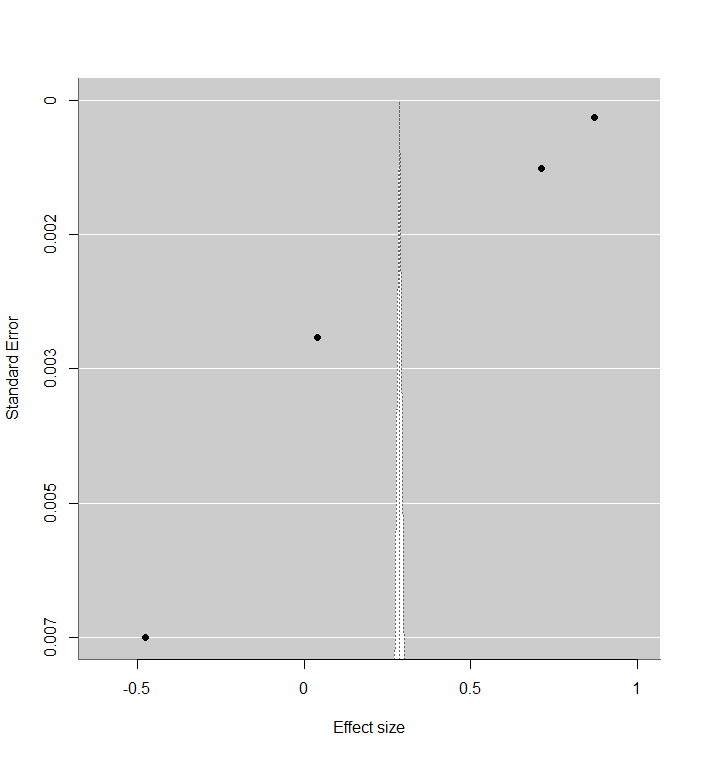*  Trim-and-fill estimated no missing studies and produced an identical pooled RR; Egger’s test indicated significant funnel-plot asymmetry (z = −6.78, p < 0.001). |
| HTN | *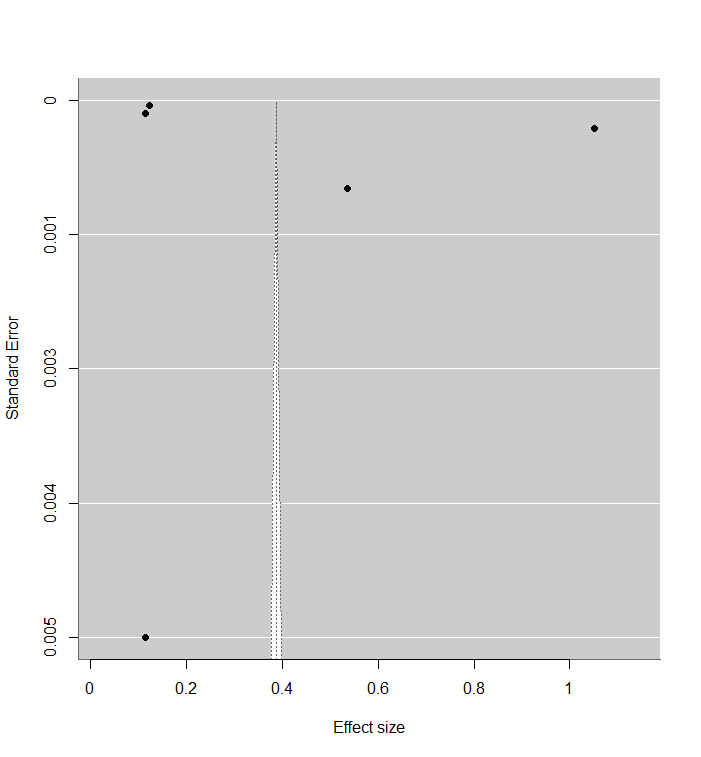*Trim-and-fill estimated no missing studies and produced an identical pooled RR; Egger’s test indicated significant funnel-plot asymmetry (z = −0.59, p = 0.56). |

- Funnel plot interpretation: with all of the meta-analyses including few studies and showing very high heterogeneity (I2 ≈ 100%), the results of the following funnel plots should be interpreted cautiously since they have low power with few studies and are proven to be unreliable when between-study heterogeneity is extreme.

With statistical funnel plot asymmetry being unreliable, we have to refer to qualitative assessment of publication bias.

- Inclusion and exclusion criteria were defined clearly before developing research algorithms.
- Trial registries were included. However, we did not put an emphasis on unpublished studies.
- Language bias was deemed negligible due to every non-English study being considered, but failing to pass the other inclusion/exclusion criteria.
- From qualitative assessment, we judge publication bias to be low.
- Risk of bias for the overall systematic review was assessed using ROBIS [4](Supplementary Figure S7)

# *
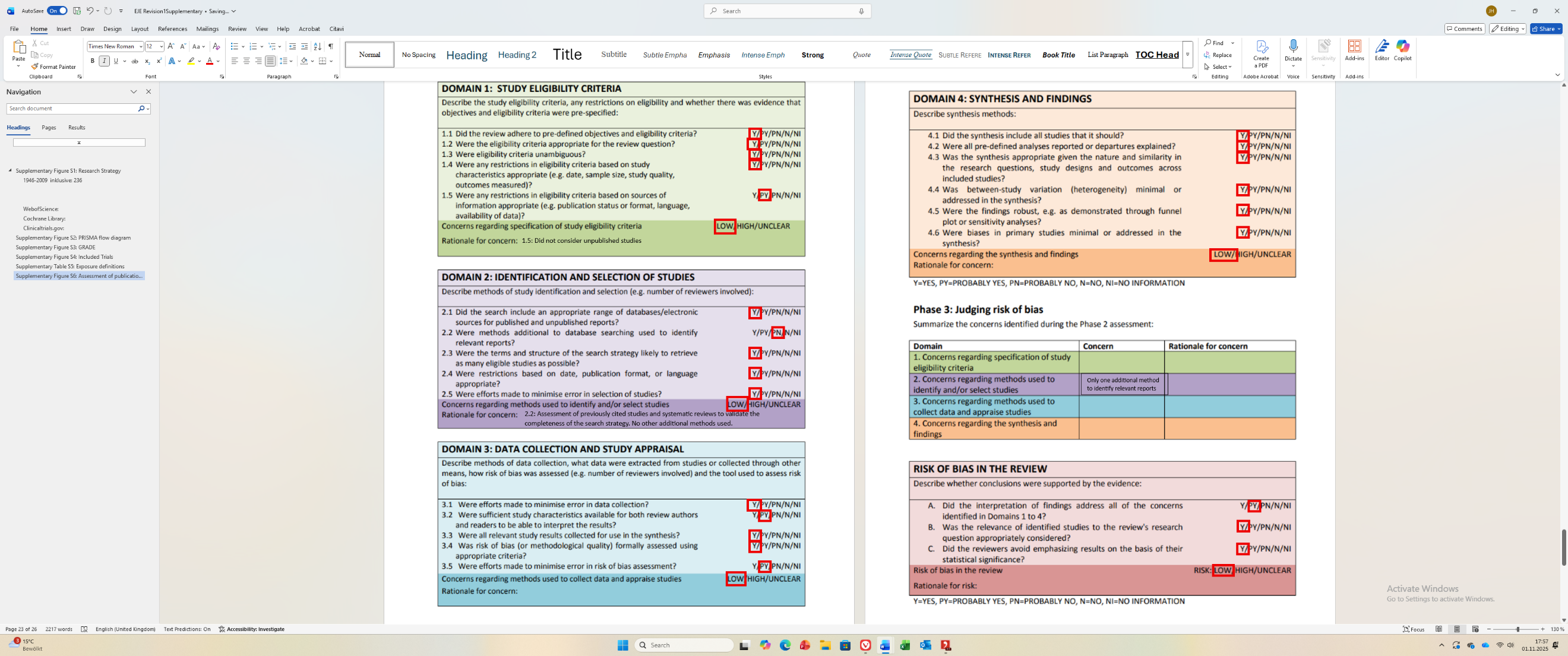
*Supplementary Figure S7: Risk of Bias assessment using ROBIS

*
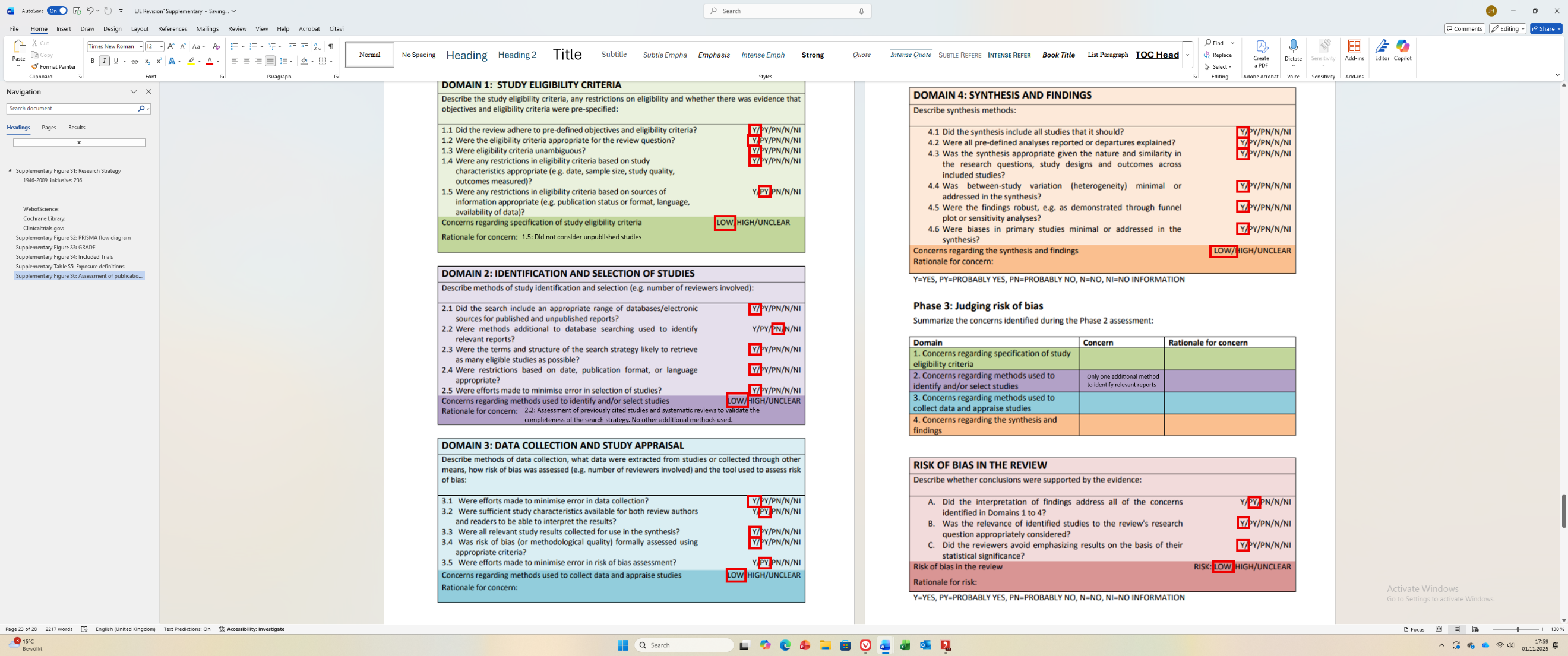
*

References

[1] Page MJ, McKenzie JE, Bossuyt PM*, et al.* The PRISMA 2020 statement: an updated guideline for reporting systematic reviews. BMJ-BRITISH MEDICAL JOURNAL 2021; 372: n71
[https://doi.org/10.1136/bmj.n71][PMID: 33782057]

[2] Zhang Y, Akl EA, Schünemann HJ. Using systematic reviews in guideline development: the GRADE approach. Research synthesis methods 2018
[https://doi.org/10.1002/jrsm.1313][PMID: 30006970]

[3] Okawa Y, Mitsuhashi T, Tsuda T. The Asia-Pacific Body Mass Index Classification and New-Onset Chronic Kidney Disease in Non-Diabetic Japanese Adults: A Community-Based Longitudinal Study from 1998 to 2023. BIOMEDICINES 2025; 13(2)
[https://doi.org/10.3390/biomedicines13020373][PMID: 40002785]

[4] Whiting P, Savović J, Higgins JPT*, et al.* ROBIS: A new tool to assess risk of bias in systematic reviews was developed. J Clin Epidemiol 2016; 69: 225–34
[https://doi.org/10.1016/j.jclinepi.2015.06.005][PMID: 26092286]
